# 451. Comparative Effectiveness of Dalbavancin Versus Standard Therapy for Staphylococcus aureus Endocarditis in People Who Inject Drugs: A Retrospective, Propensity-Matched Cohort Study Using Real-World Data

**DOI:** 10.1093/ofid/ofaf695.150

**Published:** 2026-01-11

**Authors:** Paddy Ssentongo, Silvana Ribeiro Papp, Silvana Papp, Rashmi Banjade

**Affiliations:** Penn State Health Milton S. Hershey Medical Center, Hershey, PA; UPMC, Dover, PA; Penn State Health Hershey Medical Center, Hershey, Pennsylvania

## Abstract

**Background:**

People who inject drugs (PWID) with *Staphylococcus aureus* infective endocarditis often face barriers to completing prolonged intravenous antibiotic regimens. Discharging PWID with peripherally inserted central catheters (PICCs) raises safety concerns, including potential line misuse, leading to complications and treatment failure. Dalbavancin, a long-acting lipoglycopeptide, may offer a safer, more practical alternative, but its comparative effectiveness remains uncertain.

**Figure 1.**
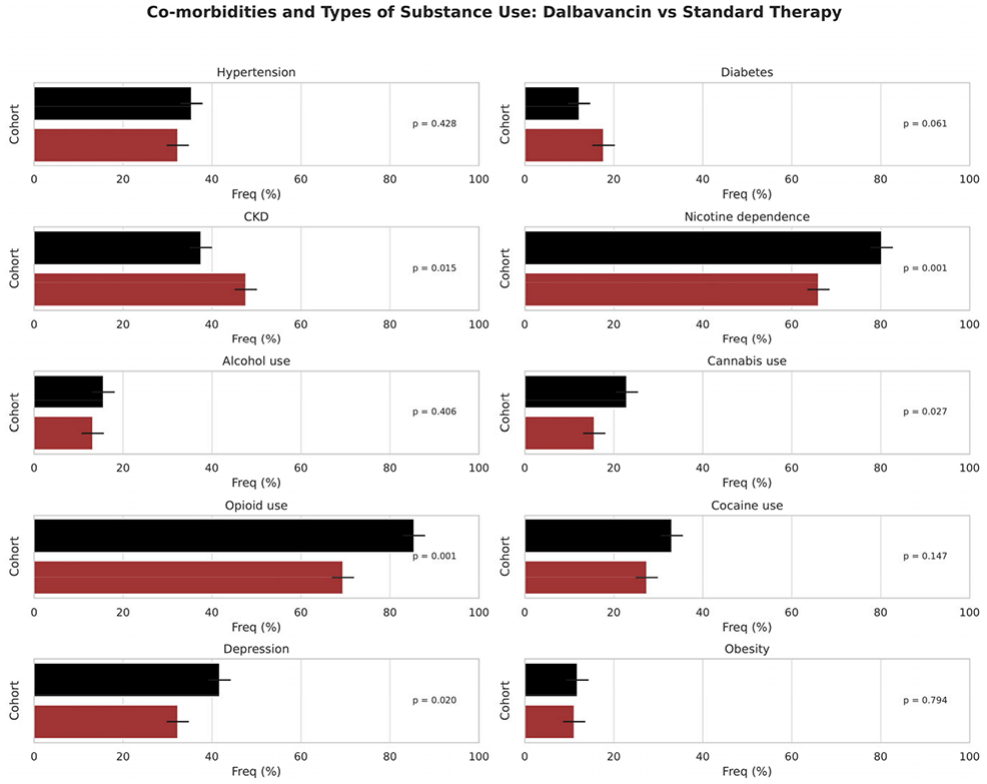
Co-morbidities and Types of Substance Use Among Patients with Staphylococcus aureus Endocarditis Receiving Dalbavancin vs Standard Therapy.

This panel illustrates the frequency (%) of key co-morbid conditions and substance use diagnoses among propensity score–matched cohorts (n=288 per group). Black bars represent patients treated with dalbavancin; red bars represent those receiving standard intravenous antibiotic therapy. Horizontal bars show group frequencies with 95% confidence intervals

**Figure 2.**
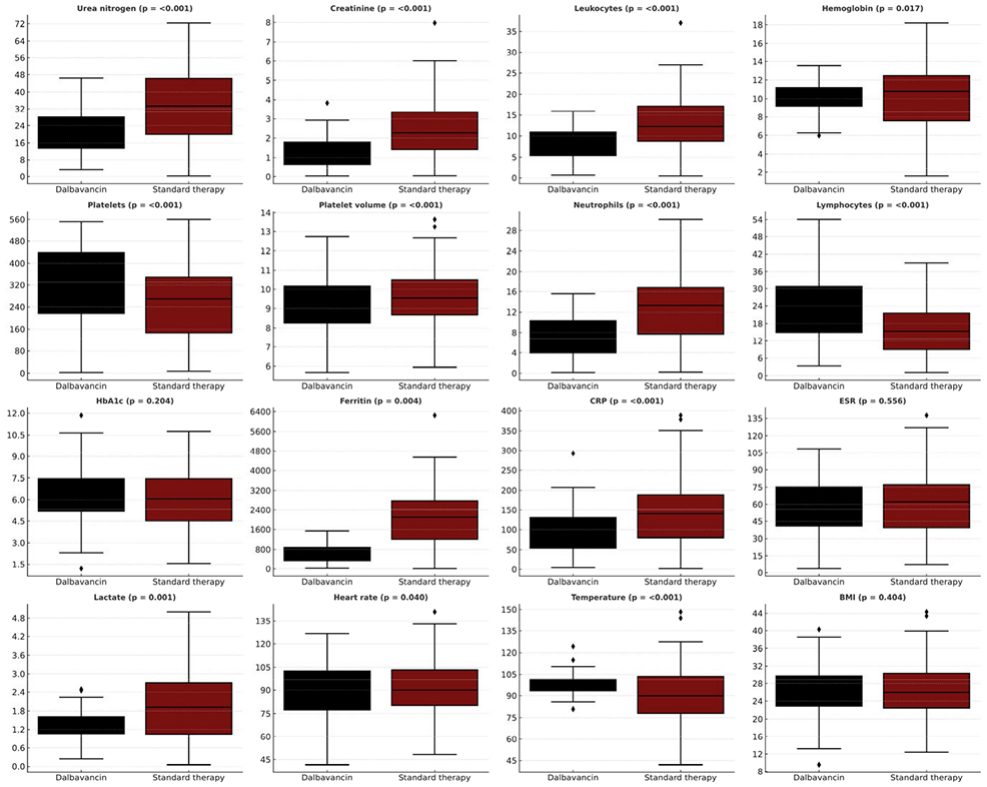
Comparison of Laboratory Values Between Dalbavancin and Standard Therapy Groups After Propensity Score Matching. Boxplots show the distribution of selected laboratory measurements for patients with endocarditis who received either dalbavancin (black) or standard therapy (dark red), following 1:1 propensity score matching. Laboratory values represent the most recent result recorded within 365 days after the index event (initiation of cohort-defining therapy

**Methods:**

Using the TriNetX Global Collaborative Network, we conducted a retrospective, real-world, propensity score–matched cohort study of adults (≥18 years) with *S. aureus* infective endocarditis and substance use history. Patients receiving dalbavancin (n=288) were matched 1:1 to those receiving standard intravenous antibiotics (vancomycin, daptomycin, cefazolin, linezolid, or nafcillin; n=288). Matching was based on age and sex. The primary outcome was 1-year all-cause mortality. Secondary outcomes included recurrent bacteremia, acute kidney injury (AKI), *Clostridioides difficile* infection, and rash.

**Figure 3.**
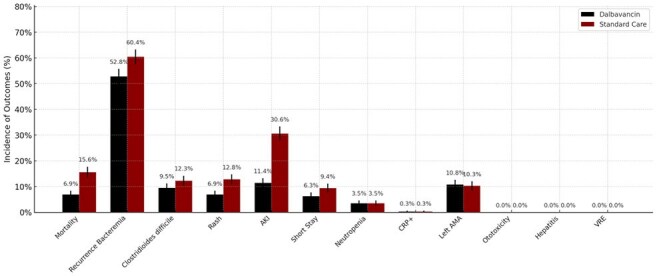
Incidence of clinical outcomes in people who inject drugs with Staphylococcus aureus infective endocarditis treated with dalbavancin versus standard therapy. Bars represent the percentage of patients experiencing each outcome, with error bars showing standard error estimates. Dalbavancin was associated with lower rates of mortality, acute kidney injury (AKI), rash, and recurrent bacteremia compared to standard intravenous antibiotics. “Left AMA” refers to patients who left the hospital against medical advice. VRE indicates vancomycin-resistant Enterococcus.

**Results:**

The median age was 38 years, and 49.3% were men. Fig 1 shows co-morbidities and types of substance use, while Fig 2 compares lab results across groups. At 1 year, mortality was significantly lower in the dalbavancin group (6.9% vs. 15.6%; risk difference −8.7 percentage points [95% CI, −13.8 to −3.6]; hazard ratio [HR], 0.44; 95% CI, 0.26–0.74; P=0.002). Dalbavancin was also associated with lower rates of AKI (11.4% vs. 30.6%; HR, 0.32; P< 0.001) and rash (6.9% vs. 12.8%; HR, 0.52; P=0.015, Fig 3). Recurrent bacteremia was less common with dalbavancin (52.8% vs. 60.4%; HR, 0.36; P=0.001).

**Conclusion:**

In this large, multicenter real-world study, dalbavancin was associated with lower mortality and fewer adverse events compared to standard therapy for *S. aureus* infective endocarditis in PWID. These findings support dalbavancin as a potentially safer and more effective alternative, especially when PICC-based outpatient therapy poses risks.

**Disclosures:**

All Authors: No reported disclosures

